# The Impact of Natural Antioxidants on the Regenerative Potential of Vascular Cells

**DOI:** 10.3389/fcvm.2019.00028

**Published:** 2019-03-22

**Authors:** Shahida Shafi, Hifzur Rahman Ansari, Wesam Bahitham, Sihem Aouabdi

**Affiliations:** King Abdullah International Medical Research Centre, King Saud Bin Abdulaziz University for Health Sciences, Ministry of National Guard-Health Affairs, King Abdulaziz Medical City, Jeddah, Saudi Arabia

**Keywords:** natural antioxidants, oxidative stress, atherosclerosis, regenerative potential, reendothelialization

## Abstract

With advances in technology, the impact of natural antioxidants on vascular cell regeneration is attracting enormous attention as many current studies are now exploring the clinical potential of antioxidants in regenerative medicine. Natural antioxidants are an important step for improving future treatment and prevention of various diseases such as cardiovascular, cancer, neurodegenerative, and diabetes. The use of natural antioxidants which have effects on several types of stem cells with the potential to differentiate into functional endothelium and smooth muscle cells (known as vascular progenitors) for vascular regeneration might override pharmaceutical and surgical treatments. The natural antioxidant systems comprise of several components present in fruits, vegetables, legumes, medicinal plants, and other animal-derived products that interact with reactive free radicals such as oxygen and nitrogen species to neutralize their oxidative damaging effects on vascular cells. Neutralization by antioxidants involves the breaking down of the oxidative cascade chain reactions in the cell membranes in order to fine-tune the free radical levels. The effect of natural antioxidants on vascular regeneration includes restoration or establishment of new vascular structures and functions. In this review, we highlight the significant effects of natural antioxidants on modulating vascular cells to regenerate vessels, as well as possible mechanisms of action and the potential therapeutic benefits on health. The role of antioxidants in regenerating vessels may be critical for the future of regenerative medicine in terms of the maintenance of the normal functioning of vessels and the prevention of multiple vascular diseases.

## Introduction

Natural antioxidants (e.g., vitamins, phenolic compounds, and carotenoids) are contained in many fruits and vegetables, whereas formulated synthetic antioxidants are additives to prevent rancidification. Natural antioxidants are present in low concentrations within cells, where they effectively reduce free radicals to provide protection system against vascular diseases. Natural antioxidants have strong potential to inhibit oxidative stress, lipid peroxidation and oxidation of breakdown products (1). They can function either individually or synergistically to remove free radicals generated during oxidative metabolism, to maintain the balance between oxidants and antioxidants. However, when there is an excessive production of reactive oxygen and nitrogen species (ROS and RNS, collectively referred to as RONS), there is then breakdown in the delicate physiological balance which results in oxidative stress. This oxidative stress eventually contributes to various chronic diseases including cardiovascular diseases, cancer, and diabetes (2). Coronary heart disease (CHD) is one of the leading causes of death and disability globally. The principal underlying cause of the disease is atherosclerosis, which occurs in large to medium size arteries, thereby leading to loss of vascular functions and cell death or necrosis (3). In addition, microvascular disorders are associated with diabetes, which is one of the main risk factors for the development of CHD. Both CHD and microvascular dysfunctions of diabetes are associated with endothelium dysfunction. Oxidative stress and inflammation are key factors responsible for endothelial dysfunction and injury, causing endothelial cells to become activated. This activation involves increased expression of several cell adhesion molecules [(CAMs) Intercellular adhesion molecule-1 (CAM-1), vascular cell adhesion molecule 1 (VCAM-1), P- and E- selectins] and chemokines (3). This initiates recruitment of monocytes/lymphocytes into subendothelium, followed by migration of dendritic and smooth muscle cells (SMCs). These cells release cytokines and growth factors, which orchestrates the process of atherosclerosis.

Both experimental animal and clinical studies show that the intake of dietary antioxidants reduces the circulating levels of the CAMs and chemokines by reducing the oxidative stress within vascular cells (4, 5). The benefit of natural antioxidants is to reduce oxidative stress directly via multiple pathways, such as regulating the formation of free radicals, the scavenging of excess radicals, and by interfering in the free radical chain reaction cascade, thereby eliminating the oxidative damage (6).

However, there is limited literature targeting the impact of natural antioxidants on the regenerative potential of vascular cells. Vascular cell regeneration potential is essentially the capability of the resident cells to accelerate the repair of the injured vascular wall in order to restore the normal vascular structure and functions. Furthermore, vascular cell regeneration is also a part of vascular senescence and growth of new blood vessels. Vascular regeneration is emerging as a clinically promising alternative strategy for repairing or preventing many vascular diseases including CHD, atherosclerosis, and microvascular complications of diabetes to improve health and quality of life. With an advent of progenitor and stem cells there is an increasing interest in novel and effective approaches to promote the regeneration of vascular cells (endothelial, vascular smooth muscle, adventitial, fibroblasts, pericytes, and progenitor/stem cells) to restore their defective functions. Resident stem and progenitor cells in the vascular wall have the ability to differentiate into vascular cell lineages. Recent studies have shown abundant stem or progenitor cells present within the vessel wall contributing to cell migration, accumulation and differentiation in the intima, and these processes not only gives rise to endothelial and SMCs, but also modulates macrophages (7, 8). Therefore, the impact of antioxidants could potentially be useful in enhancing the cellular regeneration that can modulate cellular biological processes (survival and differentiation) by counteracting the detrimental effects of ROS in various vascular disorders (9).

The aim of this review is to discuss the current information on natural antioxidants in modulating the vascular cells including the stem and progenitor cells and to provide some insights into the possible mechanism(s) by which these antioxidants restore defective cellular functions, thereby promoting the vascular regeneration process.

## Overview of Vascular Wall Cells and Their Regenerating Potential

**Figure 1:**
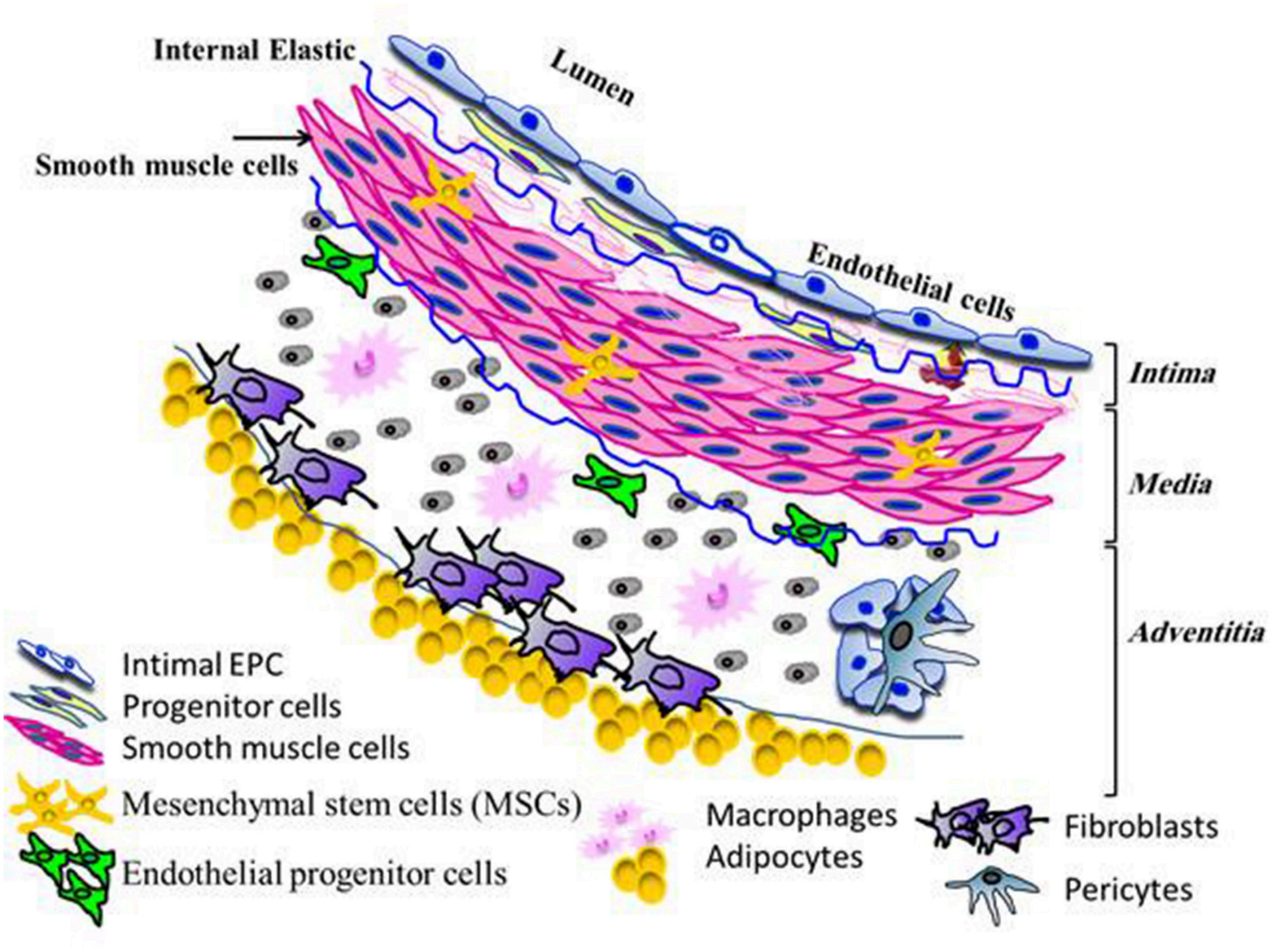
Distribution of different types of residents cells within the different layers of the normal vascular wall.

Accumulating evidence from most studies indicates an abundant number of multipotent resident stem and progenitor cells, as well as the two common distinct cell types (endothelial cells and SMCs), residing in a variety of normal vessels, including artery, vein, and microvessels (10). The vascular wall of large and medium size blood vessels comprises of three different layers, the intima, media, and adventitia (Figure 1). Strong evidence from several studies has indicated that vascular stem and progenitor cells including mesenchymal stem cells (MSCs) possess a high proliferative capacity and the potential to regenerate into functional endothelium and SMCs via various processes such as proliferation, migration, and differentiation (7, 11–15). The intima, an innermost layer, is composed of a monolayer of functional endothelial cells overlying the sub-endothelium and contains a small number of stem and progenitor cells (16–19). This layer is separated from the media layer by the internal elastic lamina. The middle layer, the media, consists of several layers of SMCs and some stem cells (7, 19). Smooth muscle cells act as scaffolds to provide stability for newly generated vessels and to regulate vascular tone. Recently, studies by Yu and co-workers on vascular repair and remodeling such as occurs in intimal hyperplasia, showed the participation of intimal SMCs and endothelial cells (7). These participating vascular cells derived from the abundant stem and smooth muscle progenitor cells, migrated from either the media or adventitia. The outermost adventitial layer is enriched with a heterogeneous population of cells such as fibroblasts and inflammatory cells (macrophages, dendritic cells, T and B cells), that are critical to the process of vascular regeneration. Beyond these cells, there are MSCs progenitor cells (Sca-1/CCR2, c-kit/CCR2) the pericytes, stem cell populations and the progenitor cells (macrophage, endothelial cells, and SMCs) (20). The stem and progenitor cells can rapidly become activated and differentiate, reflecting their role in vascular remodeling (15, 20). The vasa vasorum of the adventitia consists of a network of microvessels comprising endothelial cells enveloped by pericytes (15). The regeneration potential of the vascular wall cells in particular endothelial cells is a relatively slow process and lacks efficiency in regenerative potential (21).

A number of different natural antioxidants are being explored for their potential to promote vascular cell regeneration. This involves reprogramming of stem and progenitor cell populations to improve cell survival, differentiation, recruitment potency, and functions of vessels. Moreover, this reprogramming enhances reendothelialization and vascular remodeling. Reendothelialization is a self-repair process after injury to restore and maintain endothelium functions, through proliferation and migration of resident stem and progenitor cells as well as the adjacent endothelial cells.

## Natural Antioxidants Regulate Oxidative Stress in Vascular Cells

The vascular cells are important sites for the generation of ROS and oxidative stress. Under physiological conditions oxidative stress is compensated, however; when there is excessive production of ROS then the capacity of the antioxidant enzymes becomes inadequate to deal with such levels. This facilitates oxidative stress which can result in various types of vascular disorders (CVD, diabetes, and neurodegenerative diseases) (22, 23). The mechanism(s) responsible for this vascular damage are still not clearly known. A natural antioxidant system is present in the vascular cells as a complex defense system, which regulates oxidative stress by regulating the intracellular levels of RONS. These radicals are generated within the vascular wall cells, such as endothelial cells and SMCs, by cellular enzymes such as NADPH oxidase, xanthine oxidase, mitochondrial respiratory enzymes and uncoupled endothelial nitric oxide synthase eNOS (24). The ROS levels in fact dictate the fate of resident vascular cells. Low levels of ROS maintains the cells in their quiescent and self-renewal states, whereas at high levels they regulate multiple cell functions including intracellular signaling, proliferation, differentiation, migration (25), apoptosis (26, 27), gene expression, vascular tone, angiogenesis, and the redox potential (28). The activation of transcription factors [nuclear factor-κB, hypoxia inducible factor (HIF-1 alpha)] and eNOS stimulates cell growth and migration (29–32). However, when the antioxidant concentration becomes too low, then the activity of the repertoire of endogenous antioxidant enzymes such as glutathione peroxidase (GPx), catalase (CAT), glutathione-s-transferase (GSH), and superoxide dismutase (SOD) is reduced and this results in the breakdown of the intracellular defense barriers (33). As a consequence of this, there is an intracellular accumulation of excessively produced free radicals, triggering oxidative stress that has deleterious effects.

Recently, a number of studies have been published on the benefits of natural antioxidants, and the regulation of oxidative stress as a defined mechanism(s) in various types of vascular cells such as endothelial, VSMCs, stem, and progenitor cells (9, 34–37). Within endothelial cells and VSMCs, the oxidative stress is regulated by various pathological signaling pathways involving RONS. Indeed, animal and *in vitro* studies on the use of antioxidants (e.g., carotenoids, flavonoids, and vitamin D) suggest various intracellular mechanisms by which oxidative stress can be modulated. This includes different signaling pathways and molecules like mitogen-activated protein kinases (MAPK), transcription factor NF-kB, pro-inflammatory vascular adhesion molecules (VCAM-1, ICAM-1, E-selectin), cytokines (TNF-alpha, IL-1B, and IL-8), endogenous enzymes (SOD and CAT), inhibition of VSMC senescence, and increases in NO bioavailability (23, 34–36).

ROS levels are tightly controlled by endogenous antioxidant enzymes to reduce oxidative stress and this is critical not only in maintaining the balance between self-renewal and differentiation, but also in proliferation and apoptosis of stem and progenitor cells (9, 38). Interestingly, new scientific studies are focusing on the effects of antioxidants on reprogramming of the stem or progenitor cells. However, this reprograming of cells is also linked to oxidative stress via the increased production of ROS (39). Ji et al. in their *in vitro* study showed antioxidant supplementation not only reduced oxidative stress in the process of differentiation of the induced pluripotent stem cells (iPSCs, as a renewable source of endothelial and VSMCs), but also provided protection against DNA damage (40). On the other hand, a slight increase in ROS levels can affect the cell self-renewal process, and this effect was abolished through supplementation with combination of two different antioxidants (N-acetyl-L-cysteine and MitoQ) (41). The mechanism(s) of inhibition involved increases in GSH, H_2_O_2_, and prevention of superoxide production. Similarly, trace element antioxidants such as selenium enhanced the vascular differentiation of embryonic stem cells (ESCs) into vascular progenitor cells by reducing NADPH oxidase-4 enzyme activity, thereby lowering ROS production (42). In another *in vitro* study with adipose derived MSCs, the antioxidant supplements (*N*-acetyl-l-cysteine and ascorbic acid-2-phosphate) not only synergistically decreased oxidative stress, but also increased proliferation and cell number in the S phase of the cell cycle (43). The suggested mechanisms behind these effects involved downregulation of CDKs inhibitors and upregulation of CDK2, CDK4, and CDC2 expression.

These studies clearly show that antioxidants have beneficial effects on vascular resident cells and stem cells as potential source for vascular regeneration (differentiation, proliferation, and survival) by reducing oxidative stress through removal of ROS, and increasing the endogenous enzyme activities. The beneficial role of natural antioxidants on vascular cell regeneration in relation to their vascular functions is discussed in detail in the following sections.

## The Impact of Natural Antioxidants on the Regeneration Potential of Vascular Cells'

Several different naturally occurring antioxidants are being investigated for their potential to regenerate vascular cells and improve vascular integrity by restoring the cellular functions of diseased vessels. However, studies on natural antioxidants such as vitamins (C, D, and E) and polyphenols on the regeneration potential of vascular cells is still in its infancy. The antioxidants can be endogenous or acquired exogenously as a part of a diet or dietary supplements. Antioxidants can be classified into two main types: the enzymatic and non-enzymatic, both of which modulate free radical reactions. The non-enzymatic antioxidants include both the natural and synthetic. This article is limited to only natural antioxidants. When endogenous antioxidants are not sufficient then there is a critical need for the dietary antioxidants to maintain normal cellular functions. Natural antioxidants are contained in many fruits, vegetables, legumes, medicinal plants among others, and animal products (Table 1). The antioxidants can be further subdivided into four groups: vitamins (A, C, D, and E), carotenoids (β-carotene, lycopene, and astazanthin), polyphenols [phenolic acids (tea, honey, peach, grape seeds, and red-wine) and flavonoids (flavones, isoflavone, xanthones, and anthocyanins)] and trace elements (selenium, iron, copper, zinc, and manganese) (Table 1). The mechanisms of actions of the non-enzymatic antioxidants involves interruption of free radical chain reactions. The endogenous antioxidants include the enzymes GPx, CAT, SOD, and co-factor enzyme Q10 (oily fish, offal, and whole grain) and mitochondria-targeted antioxidant MitoQ. These endogenous defense enzymes function interactively and synergistically with diet-derived antioxidants to increase their effectiveness by either stabilizing the free radicals or by reducing their formation (66).

**Table 1:** Natural antioxidants, their mechanisms of action in regulating the oxidative stress and effects on vasculature.

**Natural antioxidants**	**Food rich in antioxidants**	**Mechanisms for oxidative stress reduction**	**Effect on vasculature**	**References**
Vitamin C (ascorbic acid)	Citrus fruits, mango, papaya, pineapple, berries, kiwis	• Scavenges ROS • Recycles α-tocopherol • Upregulates eNOS, SOD • Down regulates NADPH-oxidase	• Inhibits ox-LDL • Decreases BP • Preserves endothelial function • Reendothelialization	(44–50)
Vitamin D	Fish, egg yolk, cheese, beef liver	• Upregulates anti-oxidant enzymes • Suppresses NADPH oxidase	• Reendothelialization	(51, 52)
Vitamin E (α-tocopherol)	Vegetable oils, nuts, spinach, broccoli	• Scavenges ROS • Upregulates anti-oxidant enzymes	• Inhibits ox-LDL • Decreases blood cholesterol • Preserves endothelial function	(38, 45, 53–55)
Carotenoids β-carotene lycopene	Red, green, yellow fruits, and vegetables	• Scavenge ROS • Upregulate anti-oxidant enzymes • Decease TNFα • Increase NO • Inhibit iNOS	• Inhibit ox-LDL • Preserve endothelial function • Decrease blood cholesterol	
Polyphenols: Phenolic	Cocoa, green tea, red wine, grape seeds, peaches	• Upregulate anti-oxidant enzymes	• Inhibits ox-LDL • Decreases BP • Reendothelialization • Decrease blood cholesterol • Regulates TLR4 pathway	(6, 15, 56–58) (59–61)
Flavonoids Trace elements	Honey, berries, plants Animal dietary source	• Inhibit iNOS • Scavenge ROS	• Neovascularization	(34, 62–64) (64, 65)

*BP, blood pressure; eNOS, endothelial nitric oxide synthase; iNOS, inducible nitric oxide synthase; NADPH, Nicotinamide adenine dinucleotide phosphate; NO, Nitric oxide; ox-LDL, oxidized low-density lipoprotein; ROS, Reactive oxygen species; SOD, superoxide dismutase; TNFα, Tumor necrosis factor α; TLR4, Toll-like receptor 4*.

Natural antioxidants are currently being explored for their potential to accelerate the reendothelialization process by upregulating the adhesion molecules and stimulating rapid endothelial cell growth. Recently, an attractive new approach involving vitamins for direct reprogramming of stem or progenitor cells into endothelial cells has surfaced (44). A small number of published studies on vitamin C, D, and E demonstrated that these antioxidants promoted reendothelialization and contributed to vascular repair following vascular injury (45, 51). The reendothelialization process was the result of cell growth and proliferation (46), which improved the functions of vascular endothelial cells (45, 52, 67).

The main function of vitamins, trace elements and endogenous antioxidants is to reduce oxidative stress induced damage caused by free radicals. This occurs via mechanisms that include: inhibition of free radical formation, their decomposition and scavenging of peroxyl radicals that are converted to tocopherol radicals by α-tocopherol (vitamin E), and binding of excess free radicals to transport proteins and conversion of ROS to less reactive forms (68). Natural antioxidants may also down-regulate the cellular free radical levels by preventing the expression and activities of free radical generating endogenous enzymes (69).

Emerging evidence highlights the novel effects of natural antioxidants on modulation of vascular cells to facilitate vascular regeneration (70). However, there is still a controversy regarding synthetic antioxidant supplements (β-carotene, vitamin A, C, and E), as their use in clinical trials showed no effect on mortality (71). The reasons for the failure may have been too high doses, the exposure time was too short, different sources, and toxicity issues.

Some natural dietary antioxidants are now being recognized to play a role in the regeneration of vessels by reprogramming vascular cells. The question regarding which particular antioxidant class may be most potent in terms of their contribution in vascular cell regeneration is a challenging one at this stage.

### Vitamin C

Vitamin C (L-ascorbic acid or ascorbic acid) is an essential antioxidant available in abundance in fruits and vegetables (Table 1). Vitamin C is known to protect against vascular disorders by inhibiting the VSMC proliferation and by promoting endothelial cell proliferation in the presence of CAT enzyme and the (47, 48). Vitamin C also protects cell membranes and proteins from oxidative damage by quenching free radicals (49). This vitamin plays the role of an enzyme modulator in the vascular wall by upregulating the eNOS and SOD activities and down-regulating NADPH oxidase in the aortic wall which subsequently protects the endothelial cells (45, 50). Vitamin C also regulates vasodilation by inhibiting the effects of endothelin-1 (ET-1) and by stimulating the release of interleukin-6 (45). Recently, a new role for vitamin C has been suggested in cardiac and vascular regeneration through enhanced reprogramming of iPSCs that can differentiate into various vascular cell lineages (44). These cells have been shown to differentiate into VSMCs and endothelial cells (72).

Vitamin C may act synergistically with vitamin E to promote vessel remodeling, by modulating endothelial cell proliferation through the MAPKs activation pathway and inhibiting VSMC proliferation (49). These vitamins work by regulating the oxidative stress, via reducing redox ratio [glutathione (GSH): oxidized glutathione (GSSH)] and through MAPK and extracellular signal-regulated kinases (ERK1/2) signaling pathways (phosphorylated c-Jun NH2-terminal protein kinase, p38, and the ERK1/2). This results in the stimulation of endothelial cell growth and inhibition of VSMC growth.

### Vitamin D

Vitamin D is synthesized endogenously and can be obtained from dietary sources. Vitamin D has been shown to restore normal vascular function by reendothelialization of the damaged arterial wall (51). Vascular cells, such as endothelial cells, SMCs, and pericytes, express vitamin D receptors (VDR), but how these receptors interact with ligands to initiate cell signaling is still unknown. However, recent studies on vitamin D receptors on vascular endothelial cells of healthy volunteers, using an experimental artery injury model and VDR knockout animal models, have demonstrated that vitamin D treatment increased the number of circulating angiogenic myeloid cells (AMCs), promoted reendothelialization in the injured vessel and restored vascular endothelial dysfunction (51, 73).

These studies suggested the vitamin D in the presence of stroma cell-derived factor (SDF1) released from the vascular residential myeloid cells (macrophages or dendritic cells) promoted the migration of the AMCs to the local injury site for vascular regeneration. A meta-analysis of randomized controlled trials showed that vitamin D improved endothelial functions, despite the fact that the clinical trials were based on a small sample size (52). However, the results from clinical research and *in vitro* models published on the effect of vitamin D on angiogenesis involving endothelial cells have been contradictory with regard to the origin of the endothelial cells. These endothelial cells were either myeloid cells-derived from circulating monocytes (74) or from endothelial progenitor cells (75). Another clinical study failed to show any effect of vitamin D on endothelial cell functions (76).

### Vitamin E

Vitamin E (tocopherols and tocotrienols) is a fat soluble antioxidant found in vegetable oils such as soybean, sunflower, corn, and walnut. This vitamin exists in several forms, with α-tocopheroxyl being the most abundant form (38, 45). *in vitro* and animal experimental studies have shown that alpha-tocopheroxyl preserves endothelial integrity, inhibits VSMCs proliferation (53) and regulates endothelial function (54, 55). Vitamin E *in vitro* also inhibits monocyte-endothelial cell adhesion and aggregation, ROS monocyte, cytokine release, platelet- adhesion, and aggregation. All these effects lead to protection against the development of atherosclerosis (53).

### Carotenoids

Carotenoids are also natural antioxidants found mostly in colored fruits and green vegetables. Human beings cannot synthesize carotenoids; they are acquired from food (77). Carotenoids include β-carotene, lutein, zeaxanthin, astaxanthin, and lycopene. Although all carotenoids possess antioxidation potential (prevent oxidative damage to lipid membranes and LDL by scavenging free radicals and lowering ROS levels), some carotenoids have specific properties, for example β-carotene is a precursor of vitamin A, which has a protective role in both ocular and vascular disorders (78). Beta-carotene and lycopene are known to decrease tumor necrosis factor-α (TNF-α) mediated ROS generation at the endothelial level (62). Recent studies have shown that lycopene improved endothelial cell functions by increasing the bioavailability of NO (34). Lycopene also protects cells from oxidative stress by reducing the levels of ROS and controlling the production of antioxidants enzymes (SOD and CAT) (36, 63). Lycopene treatment of endothelial progenitor cells (EPCs) caused an increase in their proliferation, as well as reduced apoptosis and autophagy (64). These effects of lycopene are particularly important in vascular disease such as diabetes mellitus where EPCs autophagy is increased due to the increase in advanced glycation end-products.

### Polyphenols

Polyphenols are present in a wide variety of dietary food and medicinal plants, and are subdivided into two main groups: phenolic and flavonoid antioxidants (79). Recently, studies of Wang et al. *in vitro* and rat vascular graft model provided evidence that cells within the vascular wall, endothelial, SMCs, and macrophages, can be modulated by the natural polyphenolic compound resveratrol (15). This compound is present in the skin of grapes, blueberries, raspberries, mulberries, peanuts, and in red wine. This study also showed that resveratrol induced vascular stem and progenitor cell to differentiate into endothelial cells, that accelerated endothelialization of an artery via endothelial cell migration and by an increase in macrophage number (15). However, their study failed to show an inhibitory effect of resveratrol on intimal hyperplasia, whereas a previous study reported inhibition of SMC proliferation (80). This discrepancy in the inhibitory effects may have been due to the two different models being employed (vascular graft and vascular injury), where the regeneration and repair processes as well as the number of SMCs present might have been different. Evidence from other *in vitro* studies has shown that resveratrol increased proliferation and the functional activity of endothelial progenitor cells (56).

A study by Oak et al. showed that polyphenols from red wine inhibited the expression of a pro-atherosclerotic, pro-angiogenic factor, and vascular endothelial growth factor (VEGF) in VSMCs (57). This inhibition was the result of the prevention of the redox-sensitive activation of the p38 MAPK-pathway. In addition, phenolic antioxidants are known to activate vascular endothelial cells through a mechanism involving the inhibition of the nuclear transcription factor pathway (NF-kβ) (58).

### Flavonoids

Flavonoids are natural antioxidants found in abundance in seeds, citrus fruit, soya, and vegetables (81). There is limited literature available on flavonoids and their effects on vascular regeneration; however, one of the isoflavone present in legumes, genistein, can eliminate free radicals, and boost antioxidant enzyme activities when combined with 7-difluoromethoxy-5′4-dimethoxy (DFMG). This novel antioxidant compound effectively inhibits VSMCs proliferation and migration by regulating the Toll-like receptor-4 (TLR4) signaling pathway as well as reducing oxidative stress (59) and endothelial cell impairment (60). These studies suggest that genistein may possibly prevent vascular disorders by restoring vascular function and promoting vascular cell regeneration. Another strong flavonoid antioxidant, catechin, activates eNOS and regulates vascular tone by its effects on the VSMCs and has been shown to reduce the incidence of CVD in epidemiological study (61).

### Trace Elements

Trace elements (selenium, iron, copper, zinc, and manganese) are natural antioxidant components obtained from dietary sources. Selenium is a powerful antioxidant found in a variety of dietary foods; its deficiency is related to an increased risk of vascular disorders (82). The differentiation of human embryonic stem cells (ESC) to vascular progenitor cells was enhanced with selenium through ROS scavenging. The effect was associated with an increase in the ROS level, and NADPH oxidase-4 activity (9). Furthermore, these ESCs are found to be involved in neovascularization (64, 65). Dietary copper is also an essential antioxidant that has been shown to have a significant effect on vascular cell types. In an animal model of atherosclerosis, copper reduced apoptosis, increased vascular eNOS, inhibited VSMCs migration, and proliferation into the aortic intima of the artery (83).

### Mitochondrial Antioxidant (MitoQ)

Mitochondrial antioxidant (MitoQ) consists of a naturally occurring antioxidant ubiquinol attached to lipophilic cation, and has the ability to cross the plasm membrane. In a recent randomized-double blind clinical trial, the chronic administration of MitoQ to aged adults with impaired endothelial function improved vascular endothelial function by a mechanism that reduced oxidative stress in the vasculature (84). Similar effects were observed in this study with aged animals with regards to the reduction in oxidative stress. This study was a short term and it would have been interesting to see the long-term effects of MitoQ on oxidative stress by assessment of the ROS production and in a larger clinical trial.

## Health Benefits of Natural Antioxidants

Natural antioxidants have been used in health and disease to treat or prevent various vascular disorders. Natural antioxidants are being added to the diet in order to overcome their deficiency (85, 86). Indeed, health promotion authorities have encouraged a balanced diet with natural antioxidants in order to benefit from their vascular regenerating properties (87).

Despite the potential role of natural antioxidants, there is still a lack of evidence on their clinical benefits and a lack of specific molecular markers to measure the impact of dietary antioxidants on health (88). An individual's responses to natural antioxidants may depend on their genetics (89).

## Conclusions and Future Prospective

In the past, there have been vast numbers of clinical trials and animal studies published concerning the effect of natural antioxidants in vascular disease prevention. These studies failed to show natural antioxidants had any beneficial effects and the results were inconsistent with *in vitro* findings. However, findings from a small number of pre-clinical (*in vitro* and animal studies) and clinical studies discussed in this review have provided convincing evidence that natural exogenous antioxidants, including vitamins, polyphenols, and carotenoids among others, and endogenous enzymes play a critical role in regulating oxidative stress. This regulation involves elimination of excessively produced free radicals and by an increase in endogenous enzyme activities. Strong evidence from *in vitro* study showed that a combination of antioxidants exerted greater effects than the individual antioxidant on reducing oxidative stress, increasing cell proliferation and number in the S phase of the cell cycle. The underlying mechanisms responsible for these effects involved downregulation of CDKs inhibitors, resulting in upregulation of CDK2, CDK4, and CDC2 expression. Another important antioxidant MitoQ has been mentioned in passing in this review, used in a short-term small clinical study which demonstrated improvement in endothelial function by reduction in oxidative stress. Collectively, studies discussed in this review have demonstrated the mechanisms of regulation of oxidative stress and have suggested the possible involvement of multiple signaling pathways and molecules. These included transcriptional factors (e.g., NF-kβ), MAPK signaling pathways, pro-inflammatory vascular molecules, cytokines, endogenous enzymes, inhibition of VSMC senescence, and increase in NO bioavailability.

In this review, we discussed the effects of natural antioxidants on reprogramming of the stem and progenitor cells (survival, differentiation, and proliferation potentials) and their potential in vascular regeneration. This is an emerging field and investigations so far have provided strong evidence that antioxidants may be used for the regeneration of blood vessels. By regulating oxidative stress with antioxidants, the resident vascular cells such as SMCs, endothelial cells, inflammatory cells, stem and progenitor cells can be modulated to normal functional cells and to committed vascular cells. Hence, there is a need to understand the mechanisms of action of regulation of oxidative stress by natural antioxidants. Considering the prevalence of vascular disorders is increasing globally, it is imperative that these regulatory mechanisms are exploited therapeutically in order to bring about progress in the treatment of vascular disorders. More investigations are needed on the impact of antioxidants on the vascular cell regenerating potentials for future therapeutic applications.

## Author Contributions

SS carried out the literature review, created Figure 1 and wrote the manuscript. SA assisted in assembling literature sources and wrote sections on vitamin C, E, and health benefits. WB assisted in literature review and partial creation of Figure 1. HA created Table 1 and assisted in writing the section on carotenoids and the bibliography. Professor Richards Becon and Susanna Wright reviewed.

### Conflict of Interest Statement

The authors declare that the research was conducted in the absence of any commercial or financial relationships that could be construed as a potential conflict of interest.
